# Biocompatible Anisole-Nonlinear PEG Core–Shell Nanogels for High Loading Capacity, Excellent Stability, and Controlled Release of Curcumin

**DOI:** 10.3390/gels9090762

**Published:** 2023-09-18

**Authors:** Jing Shen, Jiangtao Zhang, Weitai Wu, Probal Banerjee, Shuiqin Zhou

**Affiliations:** 1 Department of Chemistry of The College of Staten Island and PhD Program in Chemistry of Graduate Center, The City University of New York, 2800 Victory Boulevard, Staten Island, NY 10314, USA; shenjingbox0225@hotmail.com (J.S.); jiangtao.zhang@cix.csi.cuny.edu (J.Z.); probalbanerjee@yahoo.com (P.B.); 2 Department of Chemistry, Yunnan Normal University, Kunming 650092, China; 3 Department of Chemistry and The Key Laboratory for Chemical Biology of Fujian Province, College of Chemistry and Chemical Engineering, Xiamen University, Xiamen 361005, China; wuwtxmu@xmu.edu.cn

**Keywords:** core–shell nanogels, nonlinear PEG, anisole, biocompatible, thermo-responsive, curcumin stability, high loading capacity, drug delivery

## Abstract

Curcumin, a nontoxic and cheap natural medicine, has high therapeutic efficacy for many diseases, including diabetes and cancers. Unfortunately, its exceedingly low water-solubility and rapid degradation in the body severely limit its bioavailability. In this work, we prepare a series of biocompatible poly(vinyl anisole)@nonlinear poly(ethylene glycol) (PVAS@PEG) core–shell nanogels with different PEG gel shell thickness to provide high water solubility, good stability, and controllable sustained release of curcumin. The PVAS nanogel core is designed to attract and store curcumin molecules for high drug loading capacity and the hydrophilic nonlinear PEG gel shell is designed to offer water dispersibility and thermo-responsive drug release. The nanogels prepared are monodispersed in a spherical shape with clear core–shell morphology. The size and shell thickness of the nanogels can be easily controlled by changing the core–shell precursor feeding ratios. The optimized PVAS@PEG nanogels display a high curcumin loading capacity of 38.0 wt%. The nanogels can stabilize curcumin from degradation at pH = 7.4 and release it in response to heat within the physiological temperature range. The nanogels can enter cells effectively and exhibit negligible cytotoxicity to both the B16F10 and HL-7702 cells at a concentration up to 2.3 mg/mL. Such designed PVAS@PEG nanogels have great potential to be used for efficient drug delivery.

## 1. Introduction

Curcumin, a natural phenolic compound derived from the turmeric plant, has attracted a great deal of attention over the past decades due to its nontoxicity, low cost, and therapeutic effects in many diseases [1,2,3]. Its ability to scavenge reactive oxygen radicals of curcumin molecules is believed to be the critical property for the prevention and treatment of diseases. For example, the antioxidant property of curcumin can play an important role in diabetes therapy [4,5,6,7,8,9,10,11,12,13,14]. Unfortunately, the exceedingly low water-solubility and rapid metabolism in the body of curcumin severely limit its bioavailability [15,16,17]. For example, only 10 nmol/L of curcumin and its metabolites were detected in plasma after patients consumed capsules with curcumin doses of 3.6 g/day for up to 4 months [18]. In another trial, 50 patients received a single intravenous dose of liposomal curcumin in the range of 10–400 mg/m^2^ over 2 h. While the dosages ≥120 mg/m^2^ already changed the red blood cell morphology, the curcumin and its metabolites in plasma became undetectable within 6–60 min [19]. In order to improve the bioavailability of curcumin, a variety of nanocarriers for curcumin have been developed [20,21,22], including polymer micelles [23,24,25,26], liposomes [27,28,29,30], inorganic nanoparticles [31,32,33], porous silica/metal–organic frames [34,35,36], biopolymer complexes/nanoparticles [37,38,39,40,41], and nanogels [42,43,44,45,46,47,48,49,50,51]. However, most of these nanocarriers developed so far exhibit a low loading capacity, which still limits the bioavailability of curcumin for clinical use. Typically, the curcumin loading capacity of these nanocarriers is in the range of 1.0–12.0 *w*/*w*% (10–120 μg curcumin per mg carriers), except for a few nanocarriers designed with a hydrophobic polystyrene and lignin core [25,49].

Responsive polymer nanogels offer great advantages in drug delivery due to their tunable size, favorable biocompatibility, interior porous network structure for incorporation and protection of therapeutics, and controllable release by specific environmental stimuli [52,53,54]. The earliest polymer nanogel system used to deliver curcumin was based on a copolymer nanogel (NanoCurcTM) from *N*-isopropylacrylamide, *N*-vinyl-2-pyrrolidone, and acrylic acid [45,46]. While curcumin loaded in this nanogel has a remarkably higher systemic bioavailability than free curcumin in an animal model, the therapeutic efficacy is still very limited due to the very low curcumin loading capacity (only 1.0–1.5 wt%) because of the hydrophilic nature of the nanogel. To introduce a dual-responsive curcumin delivery property, a variety of copolymer nanogels based on the thermosensitive poly(*N*-isopropylacrylamide) (pNIPAM) incorporated with pH-sensitive chitosan, poly(*N*,*N*-dimethylaminoethylmethacrylate) (pDMAEMA), and poly(allylamine) have been developed [48,49,50]. The incorporation of Au nanoparticles (NPs) in the polymer nanogels can induce a photothermal responsive release of curcumin [51,55]. The studies on these nanogels show that the design of the polymer materials of nanogels with specific affinity to the curcumin molecules is very important to improve the curcumin loading capacity. Our previous work shows that the increase in polystyrene domains in the nanogels can significantly enhance the curcumin loading capacity due to the hydrophobic nature of polystyrene [55]. Dinari et al. developed dual-responsive nanogels with the hyperbranched lignin NPs grafted with the crosslinked thermo-/pH- sensitive p(NIPAM-co-DMAEMA) copolymers, which show a high curcumin loading capacity because of the hydrophobic and hyperbranched nature of lignin [49]. However, the material safety in biological systems of these nanogels composed of pNIPAM or polystyrene domains is still a big concern.

Poly(ethylene glycol) (PEG) is one of the few FDA approved polymers that can be used in biomedical fields due to its nontoxicity, non-immunogenicity, and biocompatibility. To combine the thermo-sensitivity, a group of nonlinear PEG derivatives have attracted great interest for bio-applications as an alternative to the pNIPAM [56,57,58]. More importantly, the lower critical solution temperature (LCST) of these nonlinear PEG polymers, polymerized from the oligo(ethylene glycol)-methacrylate macromonomers, can be precisely tuned into the range of physiologically compatible temperatures by varying the PEG side chain lengths [56,57,58,59,60,61]. These nonlinear PEGs have been proved to be nontoxic and anti-immunogenic at relatively high concentrations (100–250 μg/mL) by FDA standards [62]. On the other hand, anisole derivatives containing phenyl methyl ether moiety are natural ingredients produced by many conifers and herbs [63]. They are important intermediates in the synthesis of fragrances, pharmaceuticals, and flavoring additives of bakery products [64]. Considering the structural similarity of the anisole moieties to the phenyl methyl ether units of curcumin molecules, we expect that the curcumin molecules will have a high affinity to the anisole moiety. Thus, a nanogel with polymer network chains composed of anisole moieties should favorably enhance the curcumin loading capacity.

**Scheme 1:**
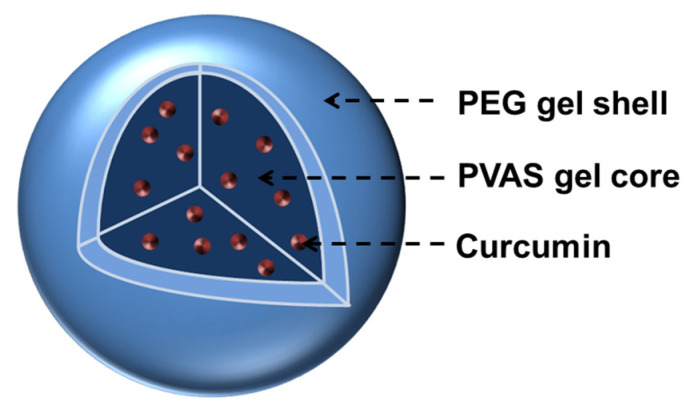
Schematic illustration of curcumin-loaded core–shell nanogels with PVAS gel as a core and the nonlinear PEG gel as a shell. The hydrophobic curcumin drug is encapsulated within the inner PVAS core, which is coated with an outer PEG gel shell to offer both stability in aqueous media and temperature sensitivity.

In this work, we design a new type of responsive core–shell nanogel as a drug nanocarrier with the aim to enhance both the curcumin loading capacity and the bio-compatibility of polymers. Specifically, we synthesize an anisole-based nanogel core from the polymerization and crosslinking of 2-vinylanisole monomers (PVAS) followed by a deposition of the thermo-responsive nonlinear PEG gel shell (Scheme 1). The hydrophobic PVAS core is designed to attract and store curcumin molecules for high drug loading capacity and the biocompatible nonlinear PEG gel shell is designed to offer temperature-responsive curcumin release. The synthetic approach for the core–shell nanogels based on stepwise precipitation polymerization in water is simple. The resultant PVAS@PEG nanogels have a spherical shape with clear core–shell morphology, narrow size distribution, controllable shell thickness, and thermo-responsive phase behavior. As expected, the PVAS@PEG nanogels display high curcumin loading capacity and thermo-controllable drug release in the physiologically relevant temperature range. Interestingly, the nonlinear PEG gel shell thickness can be used to tune the curcumin loading capacity and release kinetics. Our tests prove that the PVAS@PEG core–shell nanogels can stabilize the loaded curcumin molecules from degradation at the physiological pH of 7.4. MTT assay results show that the nanogels are nontoxic to both mouse melanoma cells B16F10 and human hepatocyte cells HL-7702 at a concentration up to 2.3 mg/mL. Confocal imaging indicates that the nanogels have good cell-penetration ability. Such rationally designed PVAS@PEG core–shell nanogels should find wide applications for the protection and delivery of other delicate hydrophobic therapeutic agents.

## 2. Results and Discussion

### 2.1. Size and Morphology of the PVAS@PEG Core–Shell Nanogels

Our strategy to prepare the PVAS@PEG core–shell nanogels involves the first synthesis of a PVAS core nanogel followed by the deposition of the nonlinear PEG gel layer on the core template, based on the well-established precipitation polymerization method. The PVAS core nanogels made from the 2-vinylanisole monomers have a hydrodynamic radius (*R_h_*) = 70 nm at 22 °C. This PVAS core is used as a template to prepare the nonlinear PEG gel shell polymerized from the comonomers of 2-(2-methoxyethoxy)ethyl methacrylate (*M*_n_ = 180 g/mol, MEO_2_MA) and oligo(ethylene glycol) methyl ether methacrylate (*M*_n_ = 300 g/mol, MEO_5_MA) and crosslinked by PEG dimethacrylate (PEGDMA, *M*_n_ ≈ 550 g/mol). In order to enable the resultant core–shell nanogel carriers to be responsive in the physiologically compatible temperature range, the molar ratio of the comonomers of MEO_2_MA: MEO_5_MA has been fixed at 1:2. Table 1 lists the size in terms of *R_h_* measured at 22 °C of the resultant PVAS@PEG core–shell nanogels (coded as VEM) prepared from the different core–shell feeding compositions. An increase in the amount of the PEG shell precursors at a fixed amount of PVAS core nanogel particles in the synthetic mixtures causes a gradual increase in the overall size of the resultant PVAS@PEG nanogel particles. The *R_h_* values of the core–shell nanogels of VEM1, VEM2, VEM3, and VEM4 are 101, 110, 138, and 165 nm, respectively. By subtracting the *R_h_* (70 nm) of the PVAS core particles, the PEG gel shell thicknesses are obtained as 31, 40, 68, and 95 nm, respectively, for VEM1, VEM2, VEM3, and VEM4 samples. This result indicates that the PEG gel shell thickness can be easily controlled by a simple change in the core–shell precursor feeding ratios in the synthesis.

**Figure 1:**
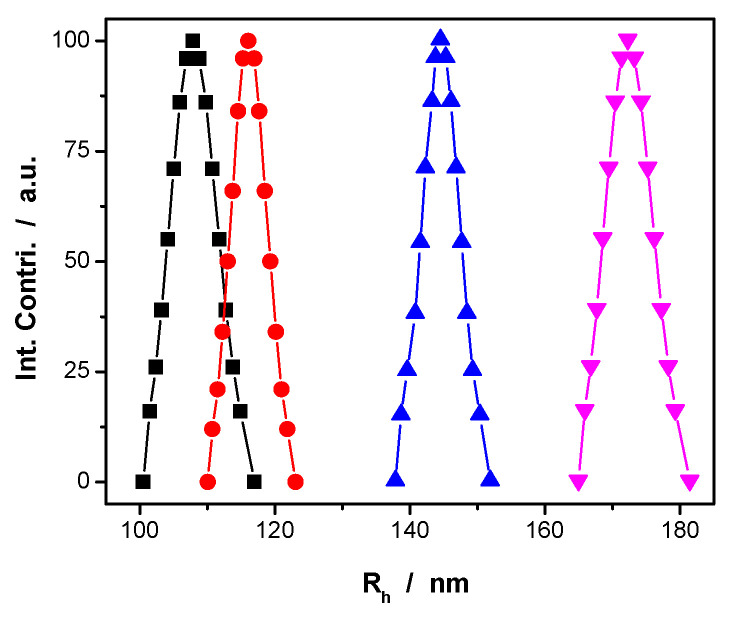
Hydrodynamic radius (*R_h_*) distribution of the PVAS@PEG core–shell nanogels of VEM1 (■), VEM2 (●), VEM3 (▲), and VEM4 (▼), measured at 22 °C and a scattering angle of θ = 45°.

Figure 1 shows the size distributions of the PVAS@PEG core–shell nanogels, in terms of the *R_h_* measured at T = 22 °C and θ = 45°, synthesized with different feeding amounts of MEO_2_MA and MEO_5_MA comonomers but at the same molar ratio of 1:2. All the obtained nanogels have a very narrow size distribution. The dynamic light scattering (DLS) measurements indicate that the nanogels obtained are nearly monodispersed with a polydispersity index of μ_2_/<Γ>^2^ = 0.001.

**Figure 2:**
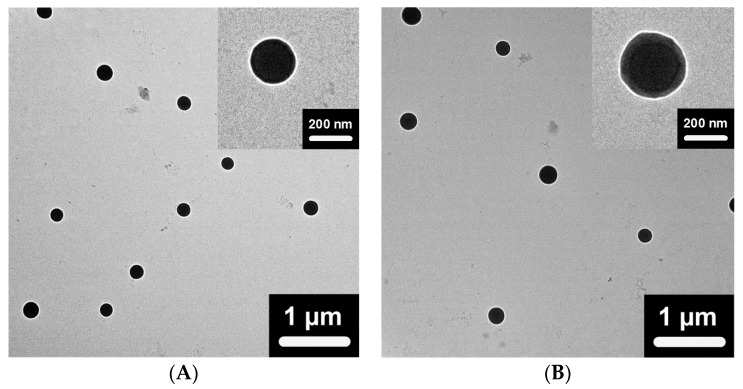
Typical TEM images of the core–shell structured PVAS@PEG nanogels VEM2 (**A**) and VEM3 (**B**).

Figure 2 shows the typical TEM images of the dried core–shell nanogels of VEM2 and VEM3. Both nanogels exhibit a spherical shape and a well-defined core–shell architecture with a clear boundary between the dark condensed core and the light contrast shell. The dark core can be attributed to the high electron density of the PVAS chains crosslinked by the short crosslinker divinylbenzene (DVB). The oligo-PEG crosslinked nonlinear PEG gel shells have a relatively low electron density and thus show a lower contrast. The clear core–shell structure indicates that the densely crosslinked hydrophobic PVAS nanogel core can hinder the hydrophilic nonlinear PEG chains of the outer shell from interpenetrating the inner core area. The TEM images also show that the shell of the VEM3 nanogels is much thicker than that of the VEM2 nanogels even though the nanogels are in a dried state, further proving that the shell thickness of the core–shell nanogels can be tuned by simply adjusting the feeding ratio of the shell precursors to the PVAS core template in the synthesis.

### 2.2. Thermo-Responsive Volume Phase Transitions of the PVAS@PEG Nanogels

**Figure 3:**
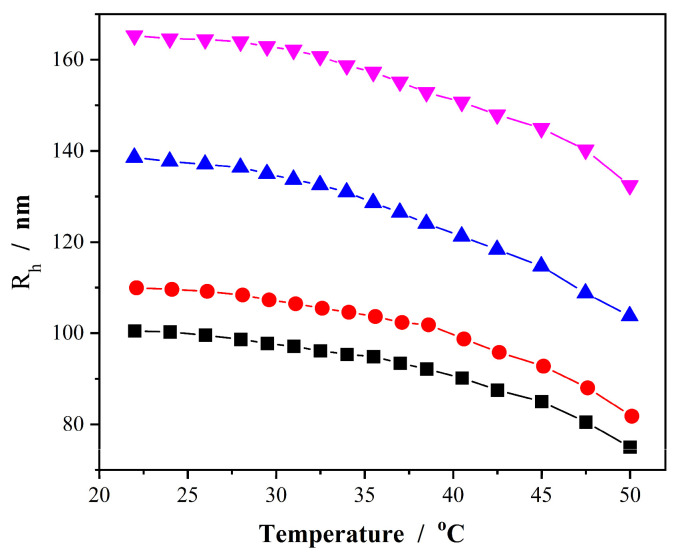
Temperature dependence of the average *R_h_* values of the core–shell structured PVAS@PEG nanogels of VEM1 (■), VEM2 (●), VEM3 (▲), and VEM4 (▼), measured at a scattering angle *θ* = 45°.

Figure 3 shows the temperature-induced volume phase transitions of the PVAS@PEG core–shell nanogels in terms of the change of *R_h_* values measured at a scattering angle of θ = 45°. Obviously, the increase in temperature of the dispersion medium can induce a significant shrinkage of the core–shell nanogels. The hydrophobic PVAS core nanogels would not undergo conformational and chemical changes in water when given external stimuli of temperature change. The observed temperature-induced volume phase transitions should be attributed to the thermo-sensitive nonlinear PEG gel shell. The nonlinear PEG network chains are composed of hydrophobic apolar carbon backbone grafted with hydrophilic PEG side chains. This counterbalance of the hydrophilic and hydrophobic forces results in the swelling/deswelling characteristic of the PEG gel shell. The critical volume phase transition temperature (VPTT) of the nonlinear PEG nanogels can be controlled by the feeding molar ratio of the two comonomers of MEO_2_MA/MEO_5_MA in synthesis [65]. As shown in Figure 3, the proper feeding molar ratio of the two macromonomers MEO_2_MA/MEO_5_MA = 1:2 in our PVAS@PEG nanogels produces a continuous shrinking in *R_h_* values across the physiologically compatible temperature range of 37–42 °C, which is a typical abnormal temperature range found in many pathological zones such as inflammation, diabetic wounds, and tumors. This temperature-responsive volume phase transition of the nanogels is reproducible and reversible, which is important for the temperature-responsive drug delivery. The results in Figure 3 also show that the nanogels do not reach a fully collapsed state at the experimental temperature window up to 50 °C, thus the nonlinear PEG gel shell is still partially swollen and hydrophilic at temperatures below 50 °C, making the PVAS@PEG core–shell nanogel particles very stable in water. Even after a few months, no sediment is observed in the nanogel solutions, which is critical for the delivery of hydrophobic drugs. While the inner PVAS core provides a hydrophobic region for the storage of hydrophobic drugs, the hydrophilic PEG gel shell can enable the nanogels to disperse in cell culture medium and further penetrate the cells. The thermo-responsive outer PEG gel shell can be utilized to regulate the transport of the drug molecules from the inner PVAS gel core region to the surrounding medium by temperature stimuli.

### 2.3. Curcumin Loading Capacity of the PVAS@PEG Nanogels

Curcumin is poorly soluble in water at acidic or neutral pHs, with the macroscopic undissolved flakes visible in the solution. On the other hand, curcumin molecules undergo a rapid hydrolytic degradation and thus lose the pharmaceutical effects when the pH is above neutral. It has been determined that the half-life for the hydrolytic degradation of curcumin in an aqueous solution containing 10% organic solvent at pH ≈ 6.0, 7.0, and 8.0 is 4.2 × 10^3^ h, 15 h, and 3.5 × 10^−2^ h, respectively. Therefore, we loaded the curcumin molecules into the PVAS@PEG core–shell nanogels at pH = 5.7, where the curcumin molecules should display no significant degradation. Based on our experience, two factors can determine the drug loading capacity for nanogel-based drug carriers. One is the affinity of the drug molecules to the polymer network chains in the gel. Another is the storage space of the nanogels for the drug molecules. In our design of the PVAS@PEG core–shell nanogels, the anisole units of the PVAS core nanogels have a similar structure to the phenyl methyl ether units in the curcumin molecules, thus the drug affinity to the PVAS network chains in the core should be very high through the π-π stacking and other association interactions, which is important to attract the curcumin molecules into the core region. However, the hydrophobic PVAS core nanogels crosslinked by small DVB molecules are in a collapsed state in the aqueous phase. To open the PVAS network of the core nanogels, the hydrophilic PEG gel shell takes on an important role. We expect that the swelling of the hydrophilic PEG gel shell will pull up the PVAS network chains in the core and thus open the pores of the PVAS core nanogels for storage of the hydrophobic curcumin molecules.

**Figure 4:**
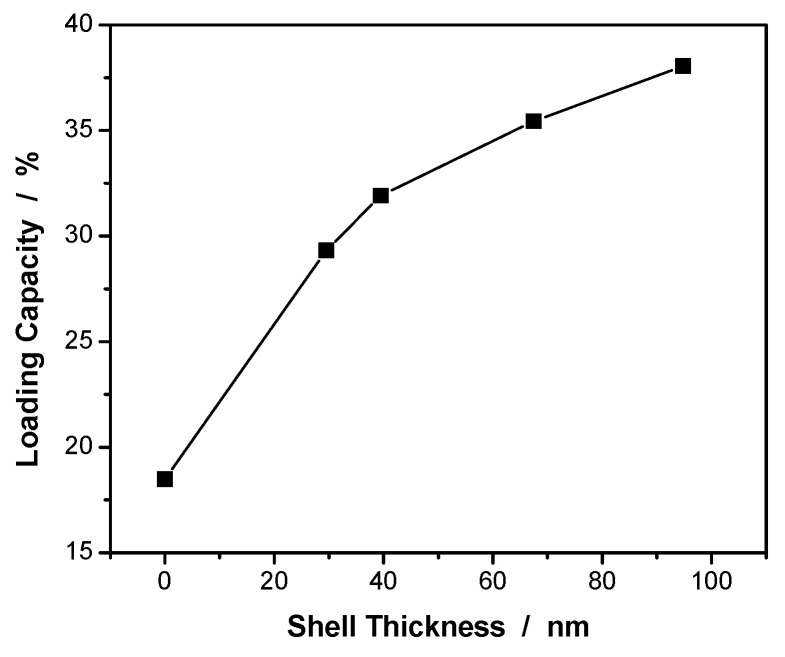
Curcumin loading capacity of the PVAS@PEG core–shell nanogels as a function of the nonlinear PEG gel shell thickness, measured at 22 °C.

Figure 4 shows the effect of the PEG gel shell thickness on the drug loading capacity of the PVAS@PEG core–shell nanogels measured at 22 °C. The curcumin loading capacity was determined to be 18.5 wt%, 29.3 wt%, 31.9 wt%, 35.5 wt%, and 38.0 wt% for the PVAS core, VEM1, VEM2, VEM3, and VEM4 nanogels, respectively. The curcumin loading efficiency was 44.9%, 71.2%, 77.5%, 86.2%, and 92.3% for the PVAS core, VEM1, VEM2, VEM3, and VEM4 nanogels, respectively. The curcumin loading efficiencies of our nanogels are comparable to those of other nanogel-based curcumin carriers [44,45,46,47,48,49,50,51]. However, the loading capacity of our core–shell nanogels are much higher than most other nanogel curcumin carriers [44,45,46,47,50,51], except a lignin-g-P(NIPAM-co-DMAEMA) nanogel with a hydrophobic lignin core [49]. Clearly, the addition of the hydrophilic nonlinear PEG gel shell onto the PVAS core can significantly enhance the curcumin loading capacity of the PVAS@PEG nanogels. The thicker the nonlinear PEG gel shell, the higher the drug loading capacity of the core–shell nanogels. Interestingly, the influence of the PEG gel shell thickness on the curcumin loading capacity has a transition point around 40 nm. When the gel shell thickness is below 40 nm, the addition of the PEG shell onto the core induces a much larger increase in the drug loading capacity. The further increase in the thickness of the PEG gel shell continuously increases the curcumin loading capacity but at a much smaller pace. This result implies that the hydrophilic PEG gel shell has limited ability to attract the hydrophobic curcumin drug molecules. Indeed, this is confirmed by the extremely low curcumin loading capacity of 1.52 wt% and loading efficiency of 3.7% for the nonlinear PEG nanogels with the same composition as the nonlinear PEG gel shell in the core–shell nanogels (See Table 1). In contrast, the PVAS core alone demonstrates a much higher curcumin loading capacity (18.5 wt%) than these nonlinear PEG nanogels because of the good affinity of the curcumin to the PVAS core network chains. This result supports that the curcumin drug molecules are mainly stored in the hydrophobic PVAS nanogel core region. Then, the question becomes why the addition of the hydrophilic PEG gel shell can further significantly enhance the curcumin loading capacity of the core–shell nanogels. To answer this question, we need to consider the influence from the pore size of the core nanogel network other than the drug affinity to the nanogel matrix. Without the hydrophilic PEG gel shell, the hydrophobic PVAS core is in a collapsed state with a small mesh size of the PVAS chain network, which has a limited space to host the curcumin drug molecules. After introducing the hydrophilic nonlinear PEG gel shell, the swollen gel shell can pull up and stretch the PVAS core network chains and thus enlarge the mesh size of the PVAS core network, although the core network chains are still hydrophobic. The swelling degree and the structure of core nanogels can be influenced by the shell gel’s property in the core–shell structured microgels. It has been observed that higher shell/core mass ratios lead to an increased expansion of the core, resulting from an elastic force developed from the swollen shell [66,67]. The open network with a large mesh size of the hydrophobic PVAS core could host many more hydrophobic curcumin molecules. The thicker the swollen nonlinear PEG gel shell, the larger the pulling force to open the hydrophobic PVAS core network, resulting in a higher curcumin loading capacity of the core–shell nanogels. When the shell thickness is increased to a certain value, the mesh size of the hydrophobic PVAS core network chains will no longer increase due to the limitation of chemical crosslinking. As such, the drug loading capacity of the core will also reach a maximum value. That is why the further increase in the thickness of a PEG gel shell larger than 40 nm would only increases the curcumin loading capacity slightly for the PVAS@PEG core–shell nanogels, because the PEG gel shell alone has a very low loading capacity for hydrophobic curcumin molecules.

### 2.4. Curcumin Stability in the PVAS@PEG Nanogels

The hydrolytic degradation of curcumin molecules occurs rapidly in the aqueous phase when the pH is above neutral [68]. Wang et al. have used high performance liquid chromatography and mass spectrometry to monitor the degradation of curcumin molecules. The results show that the curcumin molecule is initially partially deprotonated, followed by fragmentation into trans-6-(4-hydroxy-3-methoxyphenyl)-2,4-dioxo- 5-hexanal as the main product, then further decomposed to vanillin, ferulic acid, and feruloyl methane [69]. The degradation of curcumin molecules can also be monitored by photospectroscopy because the degraded products of curcumin contribute very little to the optical emission signal [70].

**Figure 5:**
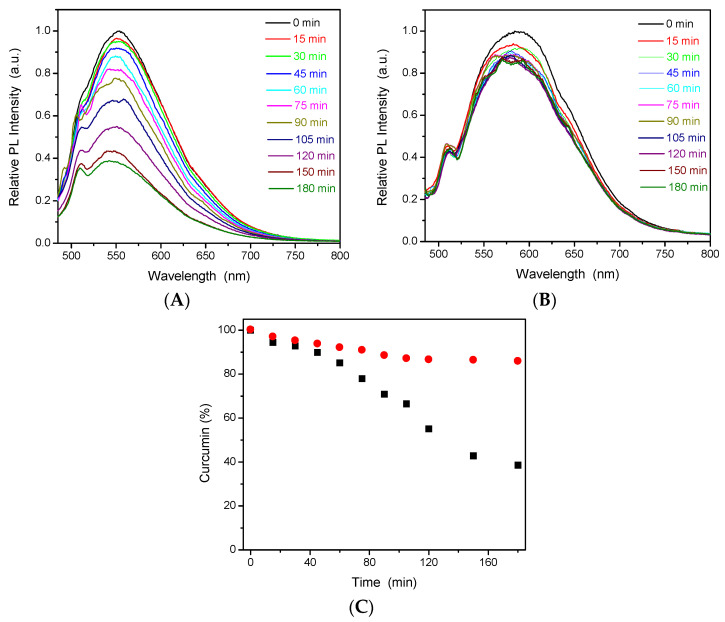
Fluorescence spectra of the free curcumin molecules (**A**) and curcumin-loaded PVAS@PEG (VEM3) nanogels (**B**) in a pH = 7.4 PBS buffer solution over a course of 180 min. The decays of the emitted fluorescence intensity at the maxima are shown in (**C**) (●—curcumin-loaded VEM3, ■—free curcumin). All experiments were carried out at 37 °C.

Figure 5 compares the fluorescence intensity change of the free curcumin molecules (A) and the curcumin molecules loaded into the PVAS@PEG core–shell nanogels (sample VEM3) (B) dispersed in a PBS solution with pH = 7.4 to the photoluminescence (PL) spectra recorded over 180 min at 37 °C and 15 min intervals. The results show that the PL intensity of the free curcumin molecules obtained at an excitation wavelength (λex) = 420 nm in PBS with pH = 7.4 at 37 °C decreases rapidly, with ~65% of curcumin molecules degrading over the 3 h course. In contrast, the PL intensity of the curcumin molecules loaded in the PVAS@PEG nanogels still remains strong, with only ~10% of curcumin molecules degrading over the initial 45 min and only about 5% of curcumin degrading over the remaining 2.5 h (Figure 5C). The initial fast degradation rate, similar to that of free curcumin molecules under the same conditions, could be attributed to those curcumin molecules adsorbed on the surface layer of the nanogels. Obviously, the curcumin molecules loaded in the inner core of the PVAS@PEG nanogels are very stable against degradation at pH = 7.4 and 37 °C, which proves that nanogel drug carriers can protect the delicate drug molecules from degradation.

### 2.5. Thermo-Responsive Curcumin Release from the PVAS@PEG Nanogels

**Figure 6:**
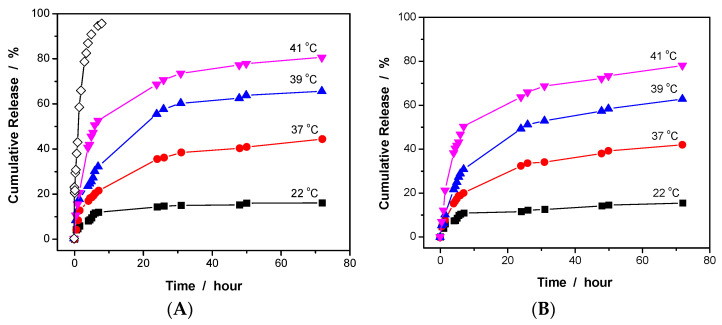

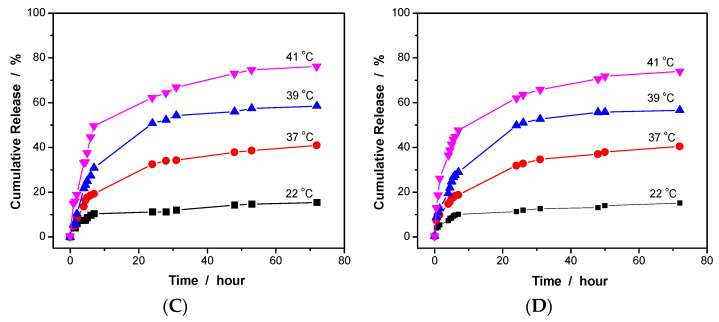
Release profiles of the curcumin (preloaded at 22 °C) from the PVAS@PEG core–shell nanogels of VEM1 (**A**), VEM2 (**B**), VEM3 (**C**), and VEM4 (**D**) at different temperatures. In the blank (◊), 1 mL diluted solution of free curcumin (containing 5% ethanol) with an equivalent amount of drug trapped in the VEM1 sample was performed at 41 °C. All release experiments were carried out in 50 mL PBS (0.005 M) with pH = 6.15.

Figure 6 shows the release kinetics of curcumin molecules from the PVAS@PEG core–shell nanogels of samples VEM1, VEM2, VEM3, and VEM4, respectively, at different temperatures. The in vitro release tests were carried out in PBS with pH = 6.15 to avoid the evident degradation of curcumin from the long-term exposure in water. A blank release experiment of free curcumin solution (containing ~5% ethanol) with an equivalent amount of drug to that loaded in the VEM1 nanogel was also performed, showing that the dialysis membrane (cutoff 12,000–14,000 Da) played a negligible role in the release kinetics (Figure 6A). Three features should be noted. Firstly, the curcumin release from the core–shell nanogels is much slower than from the free curcumin solution, indicating a sustained release of curcumin from the core–shell nanogel carriers. Secondly, the release kinetics of curcumin from the core–shell nanogels is thermo-responsive. The release of curcumin could be significantly sped up by increasing the temperature for all the VEM1, VEM2, VEM3, and VEM4 core–shell nanogels. For instance, only 16.1% of curcumin was released from VEM1 at 22 °C after 72 h. When the temperature of the dispersing medium was increased to 37, 39, and 41 °C, the percentage of curcumin released from VEM1 reached 44.4%, 65.3%, and 80.4% after the same period (72 h). This temperature dependence of curcumin release should be attributed to the thermo-responsive nonlinear PEG gel shell. The increase in temperature induced a gradual shrinking of the PEG gel shell and thus reduced the pulling force for the hydrophobic core, which in turn compressed the core space and thus squeezed out the curcumin molecules. Meanwhile, the PEG gel shell shrunk at the raised temperatures and the thinner shell thickness could also reduce the restricted diffusion path length of curcumin molecules from the core region to the medium outside of the nanogels. The higher the temperature of the releasing medium, the thinner the PEG gel shell was, which compressed more on the PVAS core, thus more curcumin molecules could be released from the core–shell nanogels. Thirdly, a slightly quicker release rate was determined at all the investigated temperatures for the PVAS@PEG core–shell nanogels with a thinner PEG gel shell. For example, the percentage of curcumin released from VEM1, VEM2, VEM3, and VEM4 at 41 °C over 72 h was 80.4%, 78.0 %, 75.9%, and 73.7%, respectively. This result could also be attributed to the factor that the thin gel shell can shorten the restricted diffusion path of the curcumin molecules from the core region to the external dispersion medium of the nanogel particles.

### 2.6. Cellular Internalization of the PVAS@PEG Nanogels

**Figure 7:**
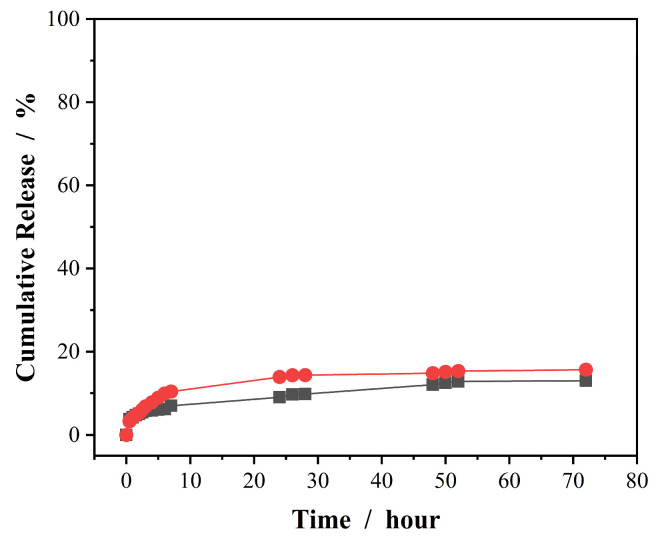
Release profiles of curcumin from the PVAS@PEG nanogels of VEM2 (●) and VEM3 (■) at 37 °C. The curcumin molecules were preloaded into the nanogels at 37 °C. All releasing experiments were carried out in 50 mL PBS (0.005 M) with pH = 6.15.

To test the cellular internalization ability of drug carriers, laser confocal imaging on cells is a good technique if the nanocarriers can display strong fluorescence or can be tagged with fluorescent objects. In our system, the free PVAS@PEG nanogels do not emit significant fluorescence in the available wavelength range of the confocal imaging equipment. On the other hand, curcumin molecules can emit strong visible fluorescence under physiological conditions. Therefore, if we load the curcumin molecules into the PVAS@PEG nanogels, the fluorescent curcumin molecules stabilized in the core region of the nanogels should enable us to detect whether the nanogels enter the cells by using laser confocal imaging. However, one challenge is to prevent the free curcumin molecules mixing with the curcumin-loaded nanogels when the cells are incubated with nanogels in the culture medium at 37 °C, because free curcumin molecules can enter the cells and illuminate the cells as well when excited by the laser. To solve this issue, we loaded the curcumin into the PVAS@PEG core–shell nanogels at 37 °C and allowed the release of curcumin molecules from the peripheral region of the nanogels for 7 days at 37 °C. As shown in Figure 7, when the curcumin molecules were loaded into the nanogels at 37 °C, only 15.6% and 12.9% of loaded curcumin was released over 72 h at 37 °C from the VEM2 and VEM3 nanogels, respectively. Moreover, the curcumin release mainly occurred in the first 10 h (from the peripheral region). After 48 h, the curcumin release was negligible. Therefore, the procedure of loading curcumin at 37 °C followed by a 72 h release can ensure that no free curcumin molecules are further released from the core–shell nanogels when cells are incubated with these curcumin-loaded nanogels in a culture medium at 37 °C. As such, only curcumin molecules still retained in the core region of nanogels emit fluorescence for cellular imaging.

**Figure 8:**
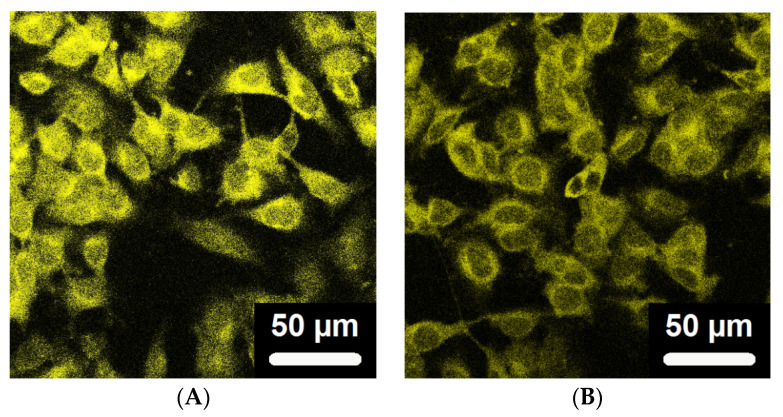

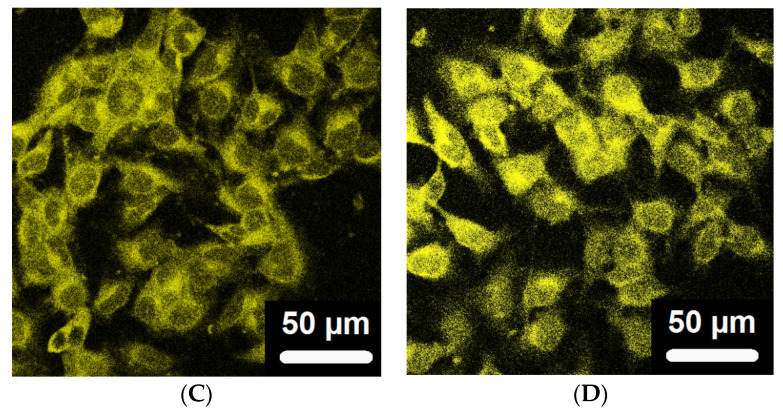
Scanning confocal fluorescence images of mouse melanoma B16F10 cells incubated with the curcumin-loaded PVAS@PEG nanogels VEM1 (**A**), VEM2 (**B**), VEM3 (**C**), and VEM4 (**D**), respectively. Excitation wavelength is 496 nm.

**Figure 9:**
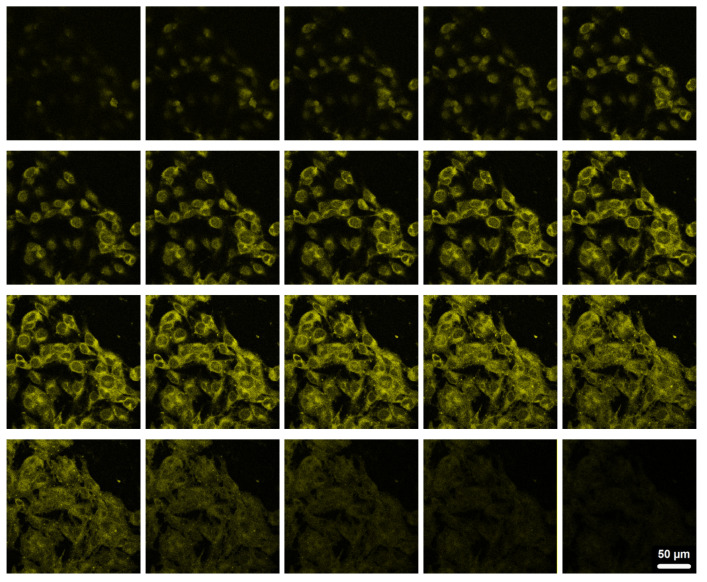
Z-Scanning confocal fluorescence transmission images of mouse melanoma cells B16F10 incubated with the curcumin-loaded PVAS@PEG core–shell nanogels (VEM3).

Figure 8 shows the scanning confocal fluorescence images of the mouse melanoma cells B16F10 after incubation with the curcumin-loaded PVAS@PEG core–shell nanogels (processed by 72 h release) for 2 h, irradiated by a laser with a wavelength of 496 nm. The bright fluorescence observed in the cells should be attributed to the curcumin molecules encapsulated inside the core–shell nanogels, because these curcumin molecules in the core region are stable with no degradation (see Figure 5). In contrast, free curcumin molecules would have degraded by ~50% over the 2 h incubation if they were present. No significant autofluorescence of cells was observed under similar conditions. To confirm that the nanogels were not just attached on the surface of cells, the top-down Z-scanning confocal fluorescence images were recorded for the B16F10 cells (Figure 9), which indicated that the curcumin-loaded PVAS@PEG core–shell nanogels indeed entered the cells and illuminated the entire cell. All the four nanogels with different PEG gel shell thickness can overcome cellular barriers and penetrate the B16F10 cells successfully. The nanogels were mainly distributed in the cytoplasm and perinuclear region of the cells. The mechanisms of endocytosis are cell-type dependent and vary between NPs with different size, charge, shape, and other surface properties [71,72]. The endocytosis process of our PVAS@PEG nanogels might be associated with the small size and specific surface properties. The nonlinear PEG gel shell is polymerized from the comonomer mixture of MEO_2_MA and MEO_5_MA in a 1:2 molar ratio. Thus, the surface of our nanogels can have some lyophilic domains mainly composed of polyMEO_2_MA. These lipophilic domains favor the interactions of the nanogels with the lipidic part of the cell membrane. These results reveal that the designed PVAS@PEG core–shell nanogels can be used for intracellular drug delivery.

### 2.7. In Vitro Cytocompatibility of the PVAS@PEG Nanogels

**Figure 10:**
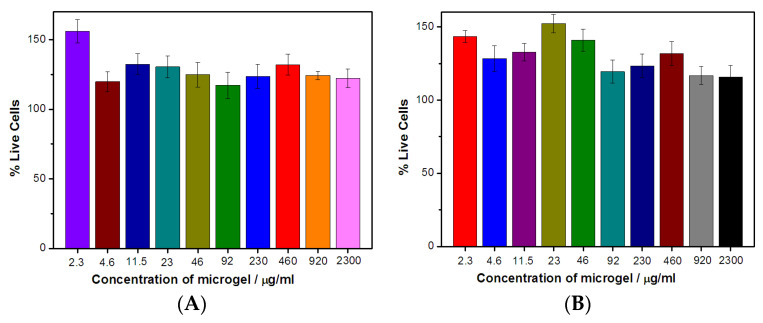
In vitro cytotoxicity results of PVAS@PEG nanogels VEM1 at different concentrations against mouse melanoma cells B16F10 (**A**) and human hepatocyte cells HL-7702 (**B**), respectively.

For biological applications, the safety of the drug carrier material is critical. To test the cytotoxicity of the PVAS@PEG core–shell nanogels, we selected two cell lines—mouse melanoma B16F10 cells and human hepatocyte HL-7702 cells—for exposure to the core–shell nanogels at different concentrations. Human hepatocyte cells play an indispensable role in metabolism, such as bile production and detoxication. Cell viability was quantified by MTT assay. Figure 10 shows the cell viability of the B16F10 cells (A) and the HL-7702 cells (B), respectively, upon treatment with the PVAS@PEG nanogels from the VEM1 sample at different concentrations. Both the B16F10 cells and the HL-7720 cells continued growing in the cell-culture medium containing the PVAS@PEG nanogels at concentrations up to 2300 μg/mL, with viability over 100% after 24 h incubation. These results indicate that the PVAS@PEG core–shell nanogels have negligible cytotoxicity against both tumor cells and normal cells. Such good biocompatibility of the newly designed nanogels is crucial for drug delivery applications, because the cells can tolerate very high concentrations of the drug carriers to deliver high doses of drug molecules in a sustained and intelligent releasing manner.

## 3. Conclusions

Well-defined thermo-responsive PVAS@PEG core–shell nanogels with the anisole-based PVAS nanogel as the hydrophobic core and the temperature-sensitive nonlinear PEG gel as the hydrophilic shell could be successfully synthesized via precipitation polymerization. Both the drug-carrier affinity and the pore (or mesh) size of the core–shell nanogels are important for enhancing the drug loading capacity. The rationally designed PVAS core chains can effectively attract, store, and protect the delicate hydrophobic curcumin molecules via π-π stacking and other association interactions, providing high drug loading capacity and stability. The hydrophilic nonlinear PEG gel shell cannot only enable the resultant core–shell nanogels dispersed very well in aqueous media but can also pull up the hydrophobic PVAS core network chains (increasing the mesh size) to further enhance the loading capacity by increasing the PEG shell thickness. The sustained release of the preloaded curcumin molecules from the core–shell nanogels can be triggered by local heat in the physiologically important temperature range. The PVAS@PEG core–shell nanogels can easily penetrate the cells. The nanogel carriers have negligible cytotoxicity against both tumor cells and normal cells in the tested concentration range up to 2.3 mg/mL, providing the ability to carry high doses of drug molecules and then release them intelligently in a sustained manner. The core–shell nanogels can be extended to load other delicate hydrophobic drugs and should find wide applications in drug delivery field.

## 4. Materials and Methods

### 4.1. Materials

2-(2-methoxyethoxy)ethyl methacrylate (MEO_2_MA, 95%), oligo(ethylene glycol)methyl ether methacrylate (MEO_5_MA, Mn = 300 g/mol), poly(ethylene glycol) dimethacrylate (PEGDMA, Mn ≈ 550 g/mol), 2-vinylanisole, divinylbenzene (DVB), 2,2′-azobis(2-methylpropionamidine) dihydrochloride (AAPH), sodium dodecyl sulfate (SDS), curcumin, anhydrous ethanol, Dulbecco’s modified eagle medium (DMEM), and fetal bovine serum (FBS) were purchased from Sigma Aldrich (St. Louis, MO, USA). MEO_2_MA, MEO_5_MA, and PEGDMA were purified with neutral Al_2_O_3_. Curcumin was purified with anhydrous ethanol. The water used in all experiments was of Millipore Milli-Q grade (Burlington, MA, USA).

### 4.2. Synthesis of PVAS Core Nanogels

The core nanogels were prepared by free radical precipitation copolymerization of 2-vinylanisole using AAPH as an initiator. A mixture of 2-vinylanisole (5.96 × 10^−4^ mol), DVB (5.61 × 10^−5^ mol), SDS (3.46 × 10^−4^ mol), and water (95 mL) was poured into a 250 mL three-neck round-bottom flask equipped with a stirrer, a nitrogen gas inlet, and a condenser. After 30 min, the temperature was raised to 70 °C and the polymerization was initiated by adding 1 mL of AAPH (0.105 M). The polymerization was allowed to proceed for 5 h. The resultant solution was centrifuged 3 times at 10,000 rpm (30 min, Thermo Electron Co. SORVALL^®^ RC-6 PLUS, Norwalk, CT, USA) superspeed centrifuge with the supernatant discarded and the precipitate redispersed in 200 mL of deionized water. The resultant PVAS nanogels were used as a core template for subsequent precipitation polymerization to add to the nonlinear PEG gel shell.

### 4.3. Synthesis of PVAS@PEG Core–Shell Nanogels

The nonlinear PEG shell precursors of MEO_2_MA and MEO_5_MA comonomer mixture in the 1:2 molar ratio and the PEGDMA crosslinker were dissolved in the 100 mL purified PVAS core nanogel dispersion. The mixture was heated to 70 °C under a N_2_ purge. After 30 min, 1 mL of AAPH (0.105 M) initiator was added to start the polymerization. The synthesis was allowed to proceed for a total of 5 h. The resultant PVAS@PEG core–shell nanogels were purified with centrifugation/redispersion in water for 3 cycles, followed by 3 days of dialysis (Spectra/Pro^®^ molecularporous membrane tubing, cutoff 12,000−14,000, Acton, MA, USA) against very frequently changed water at room temperature (~22 °C). Different feeding compositions of the core nanogels and the PEG gel shell precursors were used to control the nonlinear PEG gel shell thickness.

### 4.4. Curcumin Loading and Release

Loading: 5 mL of nanogel dispersion was stirred in an ice water bath for 30 min. Then, 4 mL of a fresh curcumin solution of 1mg/mL in anhydrous ethanol was added dropwise to the vial. After stirring overnight, the suspension was centrifuged at 10,000 rpm for 15 min at 22 °C. To remove free curcumin, the precipitate was redispersed in 5 mL of water and further purified by repeated centrifugation and washing at least six times. All the upper clear solutions were collected and the concentration of free curcumin solution was determined by a fluorescence spectrophotometer at 566 nm upon excitation at 420 nm. The optical signal was converted to a concentration based on the linear calibration curve R^2^ > 0.99, measured using the standard solutions of curcumin under the same condition. The amount of loaded curcumin in the nanogels was calculated by deducting the amount of curcumin in the upper clear solution from the total curcumin used (4 mg). The loading capacity was expressed as the mass of loaded drug per unit weight of dried nanogels. The loading efficiency was calculated by (the mass of loaded drug)/(the total mass of drug used for loading) × 100%. Loading experiments at the physiological temperature of 37 °C were also performed by using the same procedure at 22 °C.

The in vitro release of curcumin from the nanogels was evaluated by the dialysis method. The curcumin-loaded nanogel dispersion was diluted to 0.15 mg/mL for the release experiments. A dialysis bag (Spectra/Pro^®^ molecularporous membrane tubing, cutoff 12,000−14,000) filled with 1 mL of diluted curcumin-loaded nanogels was immersed in 50 mL of 0.005 M phosphate buffer solutions (PBS) with pH = 6.15 at different temperatures. The curcumin released outside of the dialysis bag was sampled at defined times and assayed by a fluorescence spectrophotometer at 566 nm upon excitation at 420 nm. Cumulative release was expressed as the total percentage of drug released through the dialysis membrane over time.

### 4.5. Internalization of Nanogels into Mouse Melanoma Cells B16F10

Round glass coverslips were placed in wells of a 24-well plate and treated with 0.1% poly-l-lysine in 100 mM PBS for 40 min. Following the treatment, the solution was aspirated and the wells were washed with PBS 3 times each. Next, B16F10 cells were plated on the glass coverslips at 80% confluence in DMEM containing 10% FBS and 1% penicillin/streptomycin. After 24 h, 500 μL of the four curcumin-loaded nanogels (0.3 μg/mL) with different PEG gel shell thickness in serum-free DMEM was added to the wells, respectively. The plate was incubated at 37 °C for 2 h. The medium was then aspirated and fresh serum-free DMEM was added to each well. Finally, the coverslips with cells were removed from the wells and mounted onto slides for confocal microscopy study.

### 4.6. In Vitro Cytotoxicity

The cytotoxicity test was performed by MTT assay, as described in our previous paper [55,73]. B16F10 cells and human hepatocyte HL-7702 cells (both 6 × 10^3^ cells/well) were cultured in DMEM containing 10% FBS and 1% penicillin/streptomycin in a 96-well plate and exposed to different concentrations of PVA@PEG core–shell nanogels, respectively. The plate was washed three times using fresh serum-free DMEM. The plate was incubated at 37 °C for 24 h. After that, 25 μL of 3-(4,5-dimethyl-2-thiazolyl)-2,5-diphenyltetrazolium bromide (MTT) solution (5 mg/mL in phosphate buffered saline (PBS)) was added to the wells. After incubation for 2 h, the solution was aspirated and 100 μL of DMSO was added to each well to dissolve the formazan crystal, and the plate was sealed and incubated overnight at 37 °C with gentle mixing. Three portions of the solution obtained from each well were transferred to the 3 respective wells of a 96-well plate. Cell viability was measured using a microplate reader at 570 nm. Positive controls contained no nanogels and negative controls contained MTT.

### 4.7. Characterization

The TEM images were taken by a FEI TECNAI transmission electron microscope (FEI, Hillsboro, OR, USA) at an accelerating voltage of 120 kV. Approximately 10 μL of diluted nanogel suspension was dropped on a Formvar-covered copper grid (300 meshes) and then air-dried at room temperature for the TEM measurements. The fluorescence spectra (or PL spectra) were, respectively, obtained by a JOBIN YVON Co. FluoroMax^®^-3 spectrofluorometer (Paris, France) equipped with a Hamamatsu R928P photomultiplier tube, with a calibrated photodiode for excitation reference correction from 200 to 980 nm and an integration time of 1 s. The pH values were obtained by a METTLER TOLEDO SevenEasy pH meter (Columbus, OH, USA). The B16F10 cells incorporated with nanogels were imaged using a confocal laser scanning microscope (LEICA TCS SP2 AOBSTM; Milton Keynes, UK) equipped with an HC PL APO CS 20 × 0.7 DRY lens. The DLS measurements were performed by a standard laser light scattering spectrometer equipped with a BI-9000 AT digital time correlator (BI-200SM, Brookhaven Instruments, Inc.; Holtsville, NY, USA). An Nd:YAG laser (150 mW, 532 nm) was used as the light source. All nanogel solutions were passed through a Millipore Millex-HV filter (Burlington, MA, USA) with a pore size of 0.80 μm to remove dust before the DLS measurements. In DLS, the Laplace inversion of each measured intensity–intensity time correlated function can result in a characteristic line width distribution G(Γ). For a purely dissuasive relaxation, Γ is related to the translational diffusion coefficient D by (Γ/q^2^)_C→0,q→0_ = D, where q = (4πn/λ)sin(θ/2), with n, λ, and θ being the solvent refractive index, the wavelength of the incident light in vacuo, and the scattering angle, respectively. G(Γ) can be further converted to a *R_h_* distribution by using the Stokes–Einstein equation, *R_h_* = (k_B_T)/(6πηD), where k_B_, T, and η are the Boltzmann constant, the absolute temperature, and the solvent viscosity, respectively [74,75].

## Figures and Tables

**Table 1:** Feeding compositions and hydrodynamic radius (*R_h_*) of the core–shell nanogels.

Sample	Core Solution (mmol)	Shell Solution (mmol)			*R_h_* (nm)
	2-Vinylanisole	MEO_2_MA	MEO_5_MA	PEGDMA	
PVAS core nanogel	0.594	––	––	––	70
PEG shell nanogel	––	0.75	1.50	2.26 × 10^−2^	––
VEM1	0.594	0.20	0.40	0.60 × 10^−2^	101
VEM2	0.594	0.25	0.50	0.75 × 10^−2^	110
VEM3	0.594	0.50	1.00	1.51 × 10^−2^	138
VEM4	0.594	0.75	1.50	2.26 × 10^−2^	165

## Data Availability

Not applicable.
